# Hydrogen peroxide production in a pilot-scale microbial electrolysis cell

**DOI:** 10.1016/j.btre.2018.e00276

**Published:** 2018-08-01

**Authors:** Junyoung Sim, Robertson Reid, Abid Hussain, Junyeong An, Hyung-Sool Lee

**Affiliations:** a Department of Civil and Environmental Engineering, University of Waterloo, 200 University Avenue West, Waterloo N2L 3G1, Ontario, Canada; b School of Civil and Environmental Engineering, Nanyang Technological University, 50 Nanyang Avenue, Singapore 639798, Singapore

**Keywords:** Hydrogen peroxide, Microbial electrolysis cells, Pilot tests, Peroxide loss, Decomposition

## Abstract

A pilot-scale dual-chamber microbial electrolysis cell (MEC) equipped with a carbon gas-diffusion cathode was evaluated for H_2_O_2_ production using acetate medium as the electron donor. To assess the effect of cathodic pH on H_2_O_2_ yield, the MEC was tested with an anion exchange membrane (AEM) and a cation exchange membrane (CEM), respectively. The maximum current density reached 0.94–0.96 A/m^2^ in the MEC at applied voltage of 0.35–1.9 V, regardless of membranes. The highest H_2_O_2_ conversion efficiency was only 7.2 ± 0.09% for the CEM-MEC. This low conversion would be due to further H_2_O_2_ reduction to H_2_O on the cathode or H_2_O_2_ decomposition in bulk liquid. This low H_2_O_2_ conversion indicates that large-scale MECs are not ideal for production of concentrated H_2_O_2_ but could be useful for a sustainable in-situ oxidation process in wastewater treatment.

## Highlights

• A pilot microbial electrolysis cell (MEC) was tested for H_2_O_2_ production.
• Passive air diffusion to a carbon electrode successfully produced H_2_O_2_.
• The highest H_2_O_2_ conversion was only 7.2%.
• Catholyte pH over 11 can mitigate H_2_O_2_ loss in MECs.

## 1 Introduction

Microbial electrochemical or electrolysis cells (MECs) are considered a potential sustainable platform for energy-efficient wastewater treatment, due to resource recovery and wastewater treatment. Because of the dual benefits, MECs have gained tremendous attention in the last decade [1,2]. Several studies have attempted pilot-scale MECs for either electricity or H_2_ production [[3], [4], [5]] to deploy MECs in field. However, none of these studies provided significant benefits of the recovered resource against input energy and materials.

H_2_O_2_-producing MECs can give significant profits over other MECs due to high cost and demand of H_2_O_2_ [6]. In addition, the recovered H_2_O_2_ from organic waste or wastewater can be used as an in-situ oxidant in wastewater treatment, improving the sustainability of wastewater management. Similar to a conventional MEC system, H_2_O_2_-producing MECs comprise of two chambers separated by an ion exchange membrane. A solution containing dissolved organic matter is fed to the anode chamber where anode-respiring bacteria (ARB) such as *Geobacter* sp., *Pseudomonas* sp., *Shewanella* sp., etc. oxidize the organics and use the anode as the electron sink [[7], [8], [9], [10]]. The electrons flow through an external circuit to the cathode where oxygen is electrochemically reduced to H_2_O_2_ at the cathode surface by the two-electron pathway shown in Eq. (1) below [11]:

(1) O_2_ + 2H^+^ + 2e^−^ → H_2_O_2_

All studies to date have examined H_2_O_2_-MECs at the lab scale, investigating H_2_O_2_ conversion efficiency, reactor design, electrode materials, and so on [[11], [12], [13], [14]]. These lab-scale experiments have commonly showed high potential of H_2_O_2_-MECs, but scale-up tests are essential to demonstrate performance and benefits of the MECs; however, no large-scale MECs for H_2_O_2_ generation have been conducted yet.

This study is the first pilot-scale MEC (110 L) experiment for H_2_O_2_ production. The pilot MEC was featured with anode modulation for provision of high surface area for biofilm formation and passive oxygen diffusion to a non-Pt carbon cathode. To evaluate the effect of catholyte pH on H_2_O_2_ yield, the pilot-scale MEC fed with acetate medium was run using an anion exchange membrane (AEM) and a cation exchange membrane (CEM), respectively, as electrode separator. Performance of the MEC was summarized, focusing on electrode potential, current density, pH, and H_2_O_2_ yield.

## 2 Materials and methods

### 2.1 Reactor configuration

**Fig. 1:**
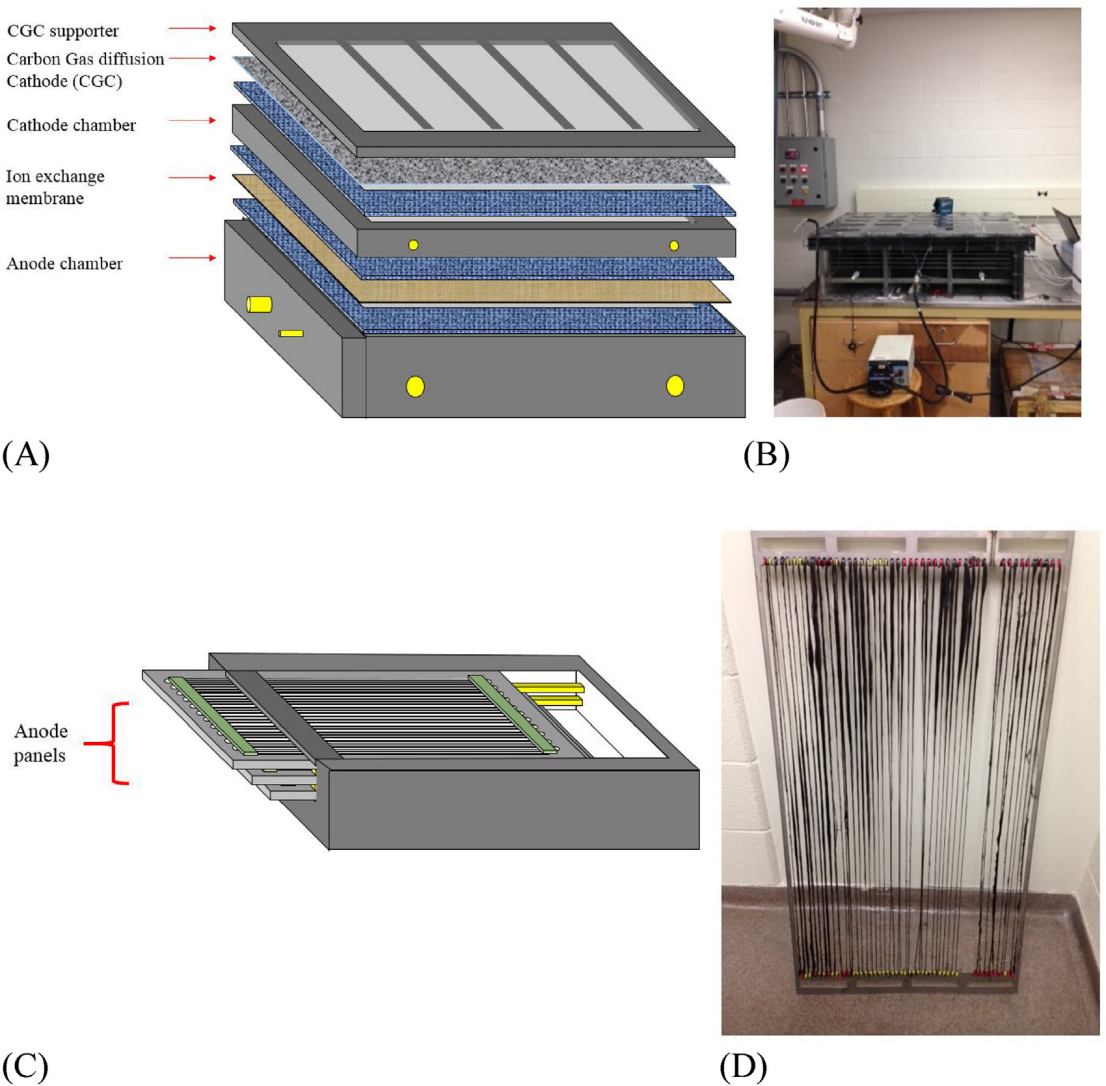
Schematic diagram of a large-scale microbial electrochemical cell (MEC). (A) MEC components, (B) photo of the MEC, (C) anode modulation, and (D) photo of an anode module.

Fig. 1 presents the schematic diagram and the picture of the pilot-scale MEC. The system has a dual-chamber configuration equipped with bioanode modules and a gas diffusion cathode (the anode chamber 1 m × 0.5 m × 0.2 m and the cathode chamber 1 m × 0.5 m × 0.02 m). The volumes of the anode and a cathode chamber were 100 L and 10 L, respectively. To provide the large surface area for biofilm formation without increasing footprint of the MEC, the anode was fabricated by connecting carbon fibers (2293-A, 24A Carbon Fiber, Fibre Glast Development Corp., Ohio, USA) to a stainless current collector, as shown in Fig. 1B. The MEC was equipped with five anode modules (Fig. 1C), providing a specific surface area of 1.27 m^2^/m^3^ anode. The carbon fibers were pretreated with nitric acid (1 N), acetone (1 N), and ethanol (1 N), and finally washed with tap water before use [15]. Peristaltic pumps (Masterflex L/S Economy Drive 7554-90, Cole-Parmer, USA) were used to circulate both anolyte and catholyte at a flow rate of 2 L/min for mixing.

Cathode catalyst selection is one of the critical parameters in H_2_O_2_ producing MECs. Precious-metal-free carbon cathodes are preferred for H_2_O_2_ production [16,17]. When using precious metal-based catalysts like platinum, the four-electron oxygen reduction to water (Eq. (2)) many outcompete the two-electron reduction to H_2_O_2_ (as shown in Eq. (1)).

(2) O_2_ + 4H^+^ + 4e^−^ → 2 H_2_O

Due to advantages of high conductivity, low cost, long-term stability and low catalytic activity of H_2_O_2_ decomposition to water, carbon-gas diffusion electrode (GD2230, Fuel Cell Earth, USA) was used as the cathode (called, carbon gas-diffusion cathode (CGC) in this study. Passive diffusion of O_2_ from atmosphere through the CGC, means no energy requirement for oxygen supply to the cathode in the MEC. An anion exchange membrane (AEM) (AMI-7001, Membranes International Inc., USA) having a surface area of 0.5 m^2^ was used for the MEC, which was later replaced with a cation exchange membrane (CEM) (CMI-7000, Membranes International Inc., USA) for comparison.

### 2.2 Inoculation and operation

The pilot MEC equipped with AEM was inoculated with effluent from lab-scale MECs (3.5 L of anolyte) operated with acetate medium, and was fed with 20 mM acetate medium [15]. The medium was sparged with ultra-pure nitrogen (99.999%) for 30 min. Then, FeCl_2_·2H_2_O (20 mM) and Na_2_S·9H_2_O (77 mM) were added to acetate medium (1 mL per L). The pH in acetate medium was constant at 7.3 ± 0.1. The cathode chamber was filled with tap water. The AEM-MEC had been run in batch mode (∼4 months) until a peak current density of ∼0.9 A/m^2^ (∼0.45 A) was repeatedly observed in the MEC. Then, experimental data was collected in the batch pilot MEC. AEM was replaced with CEM later, and the CEM-MEC was operated for comparison experiments.

To monitor voltage and electrode potentials, a saturated calomel electrode (SCE) (MF-2052, Bioanalytical System Inc. (BASI), USA) was used as the reference electrode placed in the anode chamber; here, electrode potentials were reported against SCE reference electrode. The anode modules and the cathode were connected to a data logging system (Keithly 2700, Keithley Instruments, Inc. USA) with copper wires [18]. The power supply (Array 3654A, Array Electronic co., LTD, China) was utilized as an external voltage supplier, and applied voltage was adjusted manually daily to maintain anode potential between −0.3 and −0.5 V vs SCE in which kinetically efficient ARB can be enriched well [15,19,20]. A pH probe (RK-27003-12, Cole-Parmer, USA) was installed in the anode chamber and connected to a meter (ECPHCP0550, Eutech Instruments, USA) to continuously monitor anolyte pH. For measuring catholyte pH, H_2_O_2_ concentration, and anolyte COD concentration, approximately 10 mL of liquid samples were taken.

### 2.3 Analytical method and computation

H_2_O_2_ concentration was determined spectrophotometrically with vanadate, according to the literature [21] and the H_2_O_2_ conversion efficiency was calculated using Eq. (3);

(3) $$Conversion\;\;efficiency=\frac{n\cdot F\cdot V\cdot C}{Q}$$

where n is the number of electrons transferred per mole H_2_O_2_ generated (2 mol e^−^/mol H_2_O_2_) F is Faraday’s number (96,485 C/mol e^−^), V is the catholyte volume (10 L), C is the concentration of H_2_O_2_ measured, and Q is the cumulative coulombs during operation (C).

COD measurement was carried out spectrophotometrically using the dichromate method [22]. Chemical analyses were carried out in triplicate and standard deviations were reported with average values.

## 3 Results and discussion

### 3.1 Voltage, electrode potential, current density, and COD removal

The peak current density was 0.94–0.96 A/m^2^ (0.47–0.48 A) during the experiments. This current density is much lower than ∼10 A/m^2^ in MECs fed with acetate medium, although the enrichment procedure and inoculum used in this pilot was the same to our lab scale MECs showing ∼10 A/m^2^ [23]. The significant difference in this work is the size of the MEC is several orders of magnitude larger than lab scale MECs, which suggests the importance of ARB enrichment in full scale MECs. To enrich *Geobacter* in the biofilm anode of the pilot MEC, we only used the effluent from lab-scale MECs, instead of recycle activated sludge or anaerobic digestion sludge. Despite long acclimation for ∼4 months, the maximum current density was less than 1 A/m^2^. This result means that biomass density would be very small in the biofilm anode, although five anode modules were installed to provide large surface area to ARB’s biofilm formation. This suggests the significance of inoculation or bio-augmentation in large-scale MECs.

**Fig. 2:**
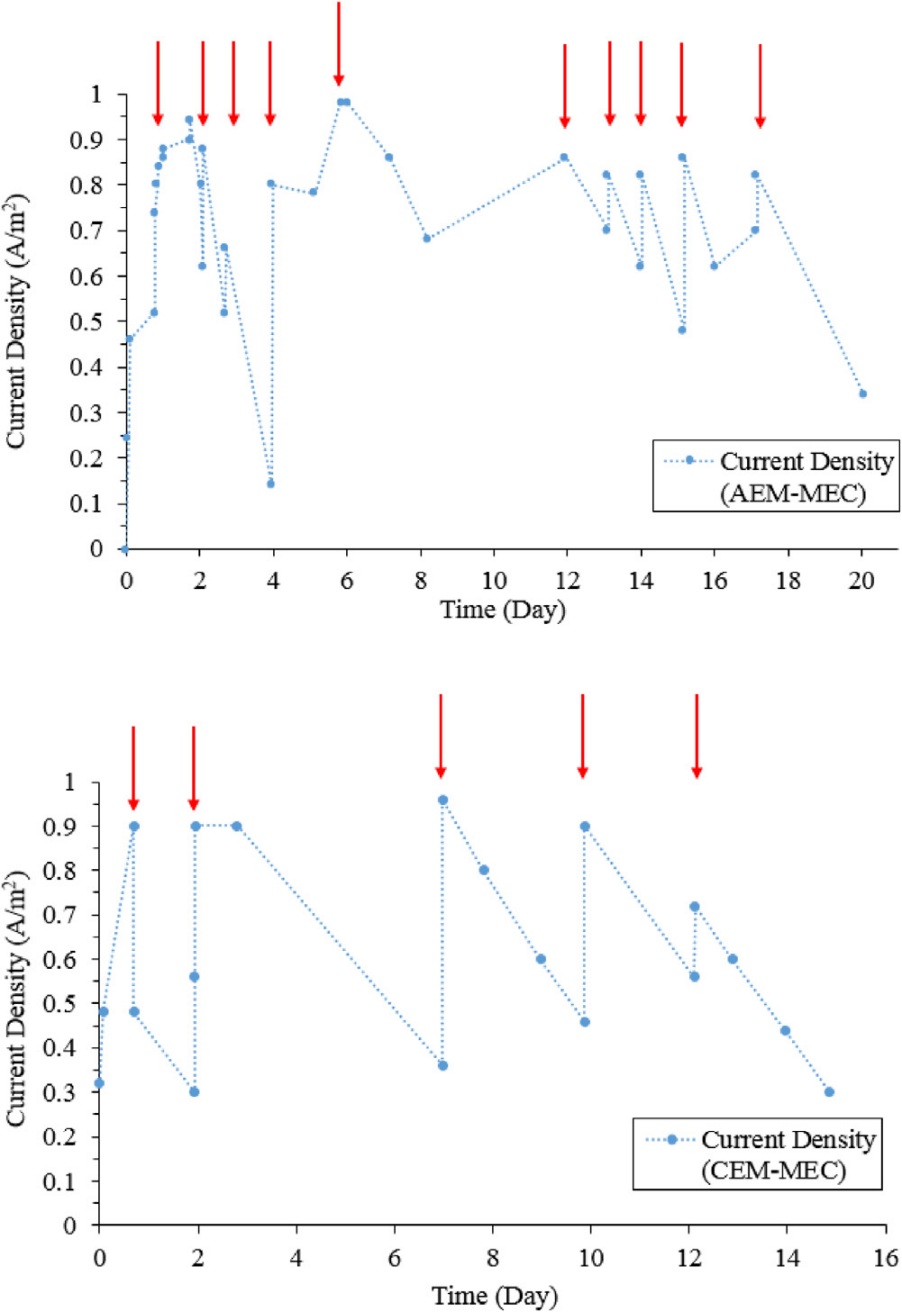
The evolution of current density in the MEC. Red arrows indicate addition of tap water into the cathode due to catholyte evaporation.

The anode potential (E_anode_) in the AEM-MEC and the CEM-MEC was kept relatively stable at −0.3 and −0.5 V (vs SCE) during operation. In comparison, significant polarization was observed for the cathode potential (E_cathode_), ranging from −2.0 to −2.4 V (applied voltage 1.6–2.0 V) and from −1.4 to −2.0 V (applied voltage 1.0– 1.6 V) in the AEM-MEC and the CEM-MEC, respectively (see Supporting information). The applied voltage is relatively higher than literature values from 0.2 to 1.3 V [11,24]. The abrupt declines in the E_cathode_ (on Day 1, 2, 3, 4, 5, 10, 12, 13, 14, 15, and 17 for the AEM-MEC, and Day 1, 2, 7, 10 and 12 for the CEM-MEC) were found, probably due to water evaporation in the cathode chamber, leading to poor contact with the cathode and consequently substantial cathodic polarization in the MEC. To overcome this operational challenge, fresh tap water was manually added to the cathode chamber. Fig. 2 demonstrates the sharp increase of current density after refilling catholyte with tap water. The CGC was installed on an exterior side of the reactor for passive air diffusion, but this study showed that in practice this cathode design can cause water evaporation in the cathode chamber and significant cathode overpotential, requiring a regular makeup of catholyte (tap water in this work). Initial COD concentration of ∼1000 mg/L was gradually decreased with time due to metabolism of ARB (Supporting information). The final COD concentration was 229 ± 1.0 mg/L and 504 ± 32 mg/L, respectively, for the AEM-MEC and the CEM-MEC at the end of batch operation (20 d for the AEM-MEC and 16 d for the CEM-MEC). Although fresh tap water was added to the anode at the end of operation (20 d and 16 d), current density was not recovered, implying anodic limitation. Accumulated acetate indicates that substrate would not account for abrupt reduction of current density in the MEC, implying other influential parameters, such as acidic pH in anolyte.

### 3.2 pH changes in anolytes and catholytes

In both AEM-MEC and CEM-MEC, the anolyte pH was gradually decreased with time; the final pH was ∼6.5 after 15–20 d of batch operation (Supporting information). This acidic pH can significantly inhibit ARB’s metabolism and decrease current density [19], which is able to account for abrupt decline of current density even in the presence of acetate at the end of batch operation (503.76 ± 32.30 mg COD/L for the AEM-MEC and 229 ± 1.0 mg COD/L for the CEM-MEC). Proton accumulation in the anode was not expected for the AEM-MEC because OH^−^ accumulated in O_2_ reduction to H_2_O_2_ can transfer from the cathode to the anode for charge neutrality in dual chamber AEM-MECs where neutral pH was kept well in the anode [11,15,25]; moles of OH^−^ accumulated in the O_2_ reduction in the cathode are equivalent to moles of protons generated from ARB’s acetate oxidation in the anode. Cathodic pH in the CEM-MEC was increased by 11.4 much higher than cathodic pH 9.7 in the AEM-MEC, which supports OH^−^ transfer from the cathode to the anode in the AEM-MEC. The acidic anolyte in the AEM-MEC implies that the OH^−^ did not neutralize all protons generated in the anode, and additional protons would be produced in the anode. The anode chamber placed on the bottom of the AEM-MEC did not have a separate gas outlet, leading to proton production from the dissolution of CO_2_ generated from ARB’s acetate oxidation (CO_2_ + H_2_O → H^+^ + HCO_3_^−^). As shown in Fig. 1, the cathode chamber was designed on the top of the MEC, forcing the anode chamber at the bottom of the MEC without gas outlets. It was challenging to design gas outlets in the anode because we could not create headspace in the anode chamber of the horizontally stacked MEC. This result indicates that MECs should be vertically stacked to have headspace in the anode chamber to mitigate anolyte acidification due to CO_2_ dissolution. In addition, ion exchange membranes can be swallowed during operation of MECs, providing small headspace in the anode. Then, biogas in the anode might be built up, accelerating membrane expansion and possibly deteriorating ion transport due to a gap between membrane and anolyte (increase of ohmic resistance). Alternatively, partial circulation of alkaline catholyte to the anode can readily neutralize acidic anolyte, but it will decrease H_2_O_2_ recovery. Catholyte circulation can be an effective solution to neutralize acidic anolyte if produced H_2_O_2_ is directly utilized to oxidize reduced forms of contaminants (e.g., BOD) in the anode chamber.

### 3.3 H_2_O_2_ concentration and conversion efficiency

**Fig. 3:**
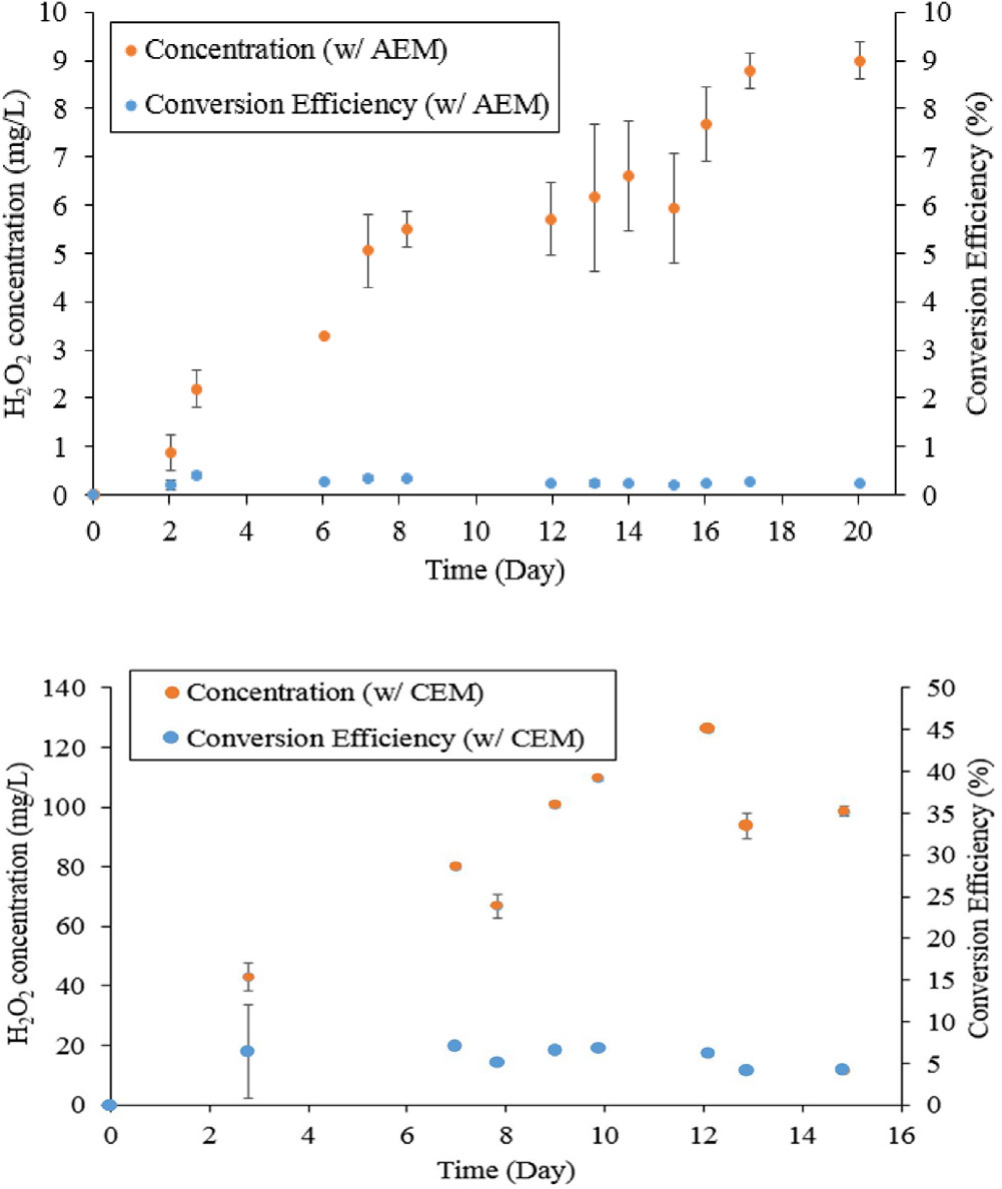
H_2_O_2_ concentration and H_2_O_2_ conversion efficiency in the MEC.

The MEC designed for passive air diffusion to the non-Pt carbon cathode successfully produced H_2_O_2_, but H_2_O_2_ production (Fig. 3) was very low as compared to literature showing high conversion of 80–90% in small-scale (<0.6 L) H_2_O_2_ MECs [12,13]. In the AEM-MEC, the cumulative H_2_O_2_ concentration was only 9.0 ± 0.38 mg/L (p = 0.007) in 20d operation, and H_2_O_2_ conversion efficiency was extremely low at 0.35 ± 0.05% (p = 0.050). For the CEM-MEC in 15d operation, the cumulative H_2_O_2_ concentration was 98.48 ± 1.6 mg/L (p = 0.007) with H_2_O_2_ conversion efficiency of 7.2 ± 0.09% (p = 0.006).

**Fig. 4:**
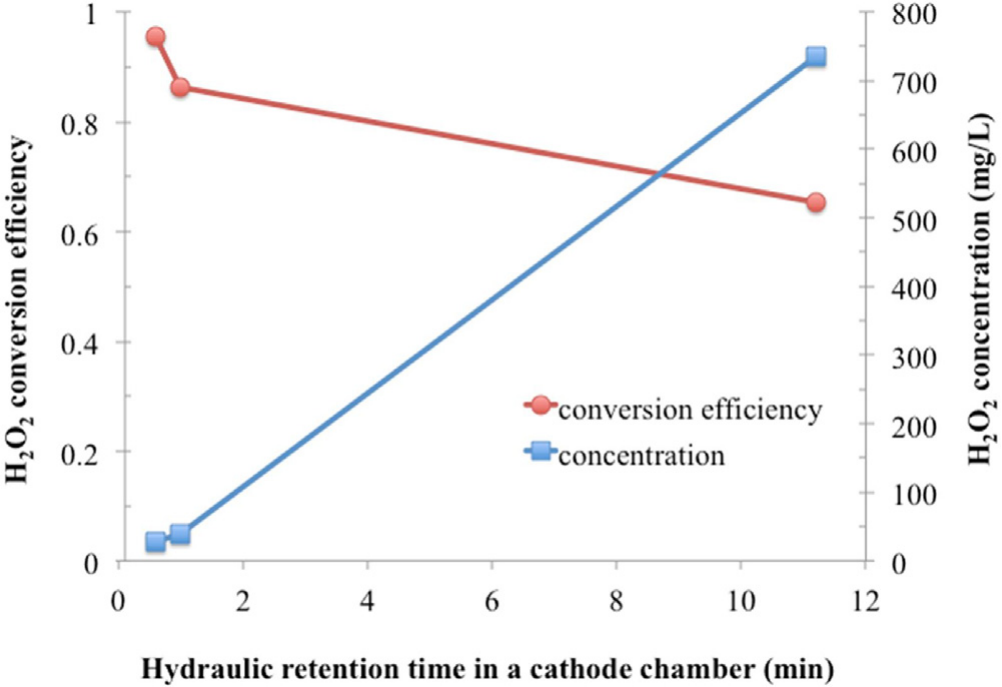
Small-scale H_2_O_2_ electrolysis cell performance. Tests were performed in a dual chamber reactor with 10 mL cathode chamber volume using 0.1 M NaCl as electrolyte and the same GDE and AEM as used in the pilot MEC. Cathode potential was fixed at −1.25 V vs SCE. Hydraulic retention time was 0.6, 1.0, and 11.2 min, respectively. The same trends were observed for the cathode potential at −1.5 to −2 V vs SCE (data not shown).

An abiotic test using a small electrolysis cell showed that H_2_O_2_ conversion efficiency approached to 100% in a catholyte continuous flow electrolysis cell as hydraulic retention time in a cathode chamber was changed from 10 to 0.6 min (Fig. 4). This supplementary test suggests that H_2_O_2_ would be formed at the cathode first and then either lost by further reduction to H_2_O at the cathode or by H_2_O_2_ decomposition in the bulk liquid. This study did not investigate which mechanism mainly account for H_2_O_2_ loss, but clearly presents that MECs are poor for recovery of concentrated H_2_O_2_. Preventing significant, spontaneous H_2_O_2_ loss seems very challenging in large MECs, and hence it is efficient to utilize the H_2_O_2_ generated from the cathode immediately, such as in-situ oxidation. The higher pH 11.4 in the CEM-MEC mitigated H_2_O_2_ losses, probably because of H_2_O_2_ ionization to HO_2_^−^ (pK_a_ = 11.65); the electrostatic repulsion between the peroxide species and the cathode can attenuate the H_2_O_2_ loss on the cathode [26].

## 4 Conclusions

This study first assessed H_2_O_2_ production in the pilot MEC equipped with the non-Pt cathode for passive air diffusion. The non-Pt carbon cathode successfully produced H_2_O_2_ without intensive air supply, but the maximum cumulative H_2_O_2_ concentration was only 98 mg/L in 20d of operation with 7.2% of conversion efficiency, indicating significant losses of H_2_O_2_ in either further reduction on the cathode or decomposition in bulk liquid. It is challenging to stop spontaneous, substantial H_2_O_2_ losses in the pilot MEC, and hence using H_2_O_2_-MECs as in-situ oxidation will be more practical than H_2_O_2_ synthesis.

## Conflict of interest

None.
