# Supplementary material for: Hydrogen peroxide production in a pilot-scale microbial electrolysis cell
Source: Biotechnol Rep (Amst). 2018 Aug 1;19:e00276. doi: 10.1016/j.btre.2018.e00276 (PMC6127372; doi:10.1016/j.btre.2018.e00276)
Supplement: Supplementary file 1 [file mmc1.docx]

Supporting Information

# **Hydrogen peroxide production in a pilot-scale microbial electrolysis cell**

Junyoung Sim, Robertson Reid, Abid Hussain, Junyeong An, Hyung-Sool Lee^*^

Department of Civil and Environmental Engineering, University of Waterloo, 200 University Ave. W., Waterloo N2L 3G1, Ontario, Canada

*Corresponding author: Phone: +1-519-888-4567 Ext. 31095; Fax: +1-519-888-4349; E-mail: [hyung.will.lee@gmail.com](mailto:hyung.will.lee@gmail.com)


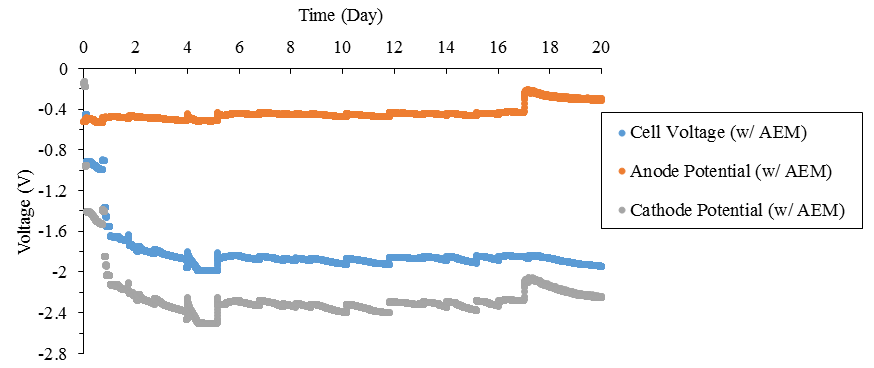


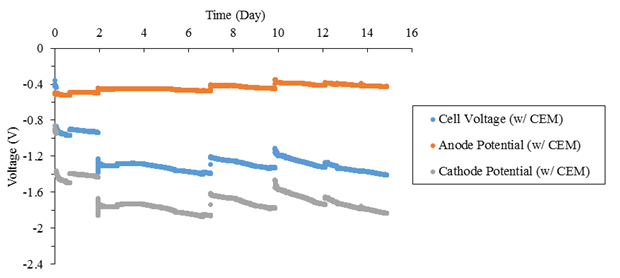


Figure S1. Cell voltage and electrode potentials in the AEM-MEC and the CEM-MEC.


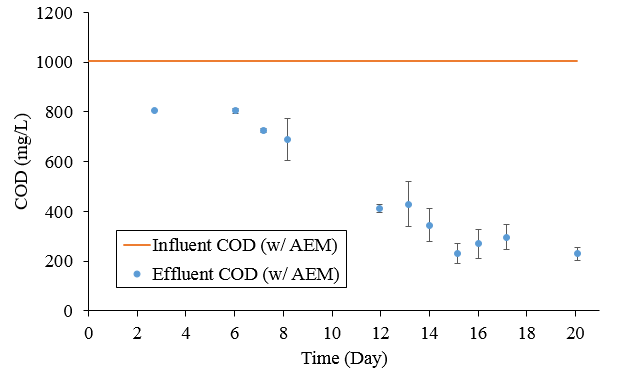

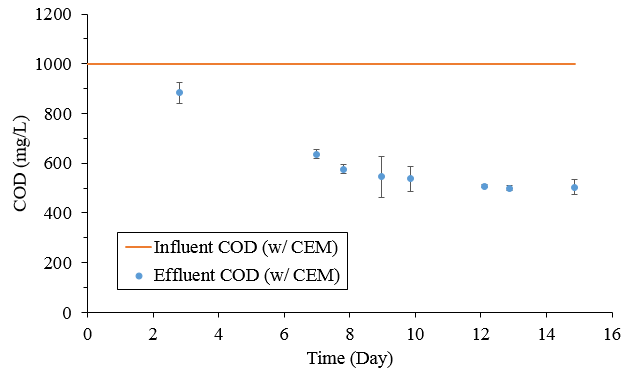


Figure S2. The evolution of COD concentration with time in the MEC.


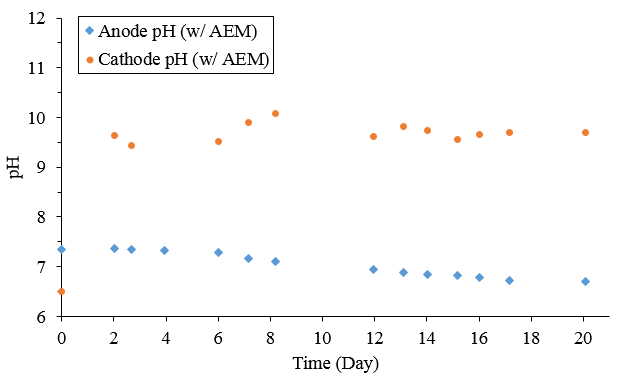

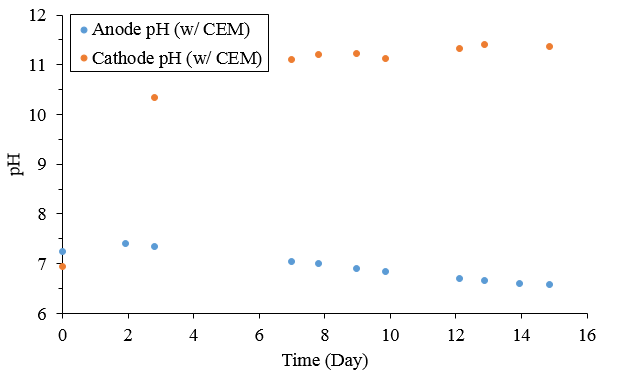


Figure S3. pH trends of anolyte and catholyte in the MEC.
